# Major Adverse Cardiovascular Events: An Inevitable Outcome of ST-elevation myocardial infarction? A Literature Review

**DOI:** 10.7759/cureus.5280

**Published:** 2019-07-30

**Authors:** Ishan Poudel, Chavi Tejpal, Hamza Rashid, Nusrat Jahan

**Affiliations:** 1 Internal Medicine, Department of Research, California Institute of Behavioral Neurosciences and Psychology, Fairfield, USA; 2 Family Medicine, Department of Research, California Institute of Behavioral Neurosciences and Psychology, Fairfield, USA; 3 Internal Medicine: Critical Care, Department of Research, California Institute of Behavioral Neurosciences and Psychology, Fairfield, USA

**Keywords:** stemi, stemi major adverse cardiovascular events, stemi complications, stemi review

## Abstract

Major adverse cardiovascular events (MACE) remain the major cause of mortality and morbidity in patients with STEMI (ST-elevation myocardial infarction). The current literature is aimed to analyze the occurrence of MACE following STEMI irrespective of treatment provided, and follow up after the first diagnosis of STEMI. A PubMed search for Studies of STEMI identified 24,244 articles. After applying our inclusion/exclusion criteria, we found out 75 articles of relevance wherein MACE and its components were considered to be the primary endpoint. These 75 articles included eight Cohort Studies, 13 clinical trials including five randomized controlled trials (RCT), one case-control Study, one cross-sectional study, one review article, and 51 other observational studies. Our analysis shows that MACE remains one of the strongest adverse outcomes among STEMI patients. The current literature review found out the incidence of MACE was 4.2 % to 51% irrespective of the mode of treatment, and follow-ups lasting up to 10 years from the time of STEMI diagnosis.

## Introduction and background

ST-elevation myocardial infarction (STEMI) has multiple definitions proposed over time and most of them can inclusively be defined as symptoms of ischemia of the myocardium that presents with the classical electrocardiographic change of elevation of ST-segment at J point and positive cardiac biomarkers above the accepted blood level [1]. Electrocardiographically STEMI can be defined as ST-elevation (STE) of ≥ 1 mm at the J point in 2 contiguous chest and limb leads excluding V2-V3, which must be ≥ 2 mm in men or ≥ 1.5 mm in women [2].

Major adverse cardiovascular events (MACE) has no concrete definition, but over time various definitions have been used in cardiovascular research with MACE selected as primary or secondary end-point. It has been defined by various authors since mid-1990 to include an overlapping range of adverse events [3,4]. Multiple adverse events included in different research as a component of MACE are heart failure, non-fatal re-infarction, recurrent angina pain, re-hospitalization for cardiovascular-related illness, repeat percutaneous coronary intervention (PCI), coronary artery bypass grafting and all-cause mortality [5]. MACE can also include unscheduled coronary revascularization, stroke, re-infarction and all-cause death and mortality [6]. MACE with myocardial infarctions have been assessed in the past and multiple articles have been published regarding specific percentage of patients having MACE after particular medical management or after undergoing certain procedures (like PCI) for both STEMI and NSTEMI. The aim of the study is to quantify the available data on the risk of MACE in patients with STEMI irrespective of the mode of management.

## Review

Method

Literature was searched in PubMed with parallel strategies based on MeSH subheadings and regular keywords for data collection. Table 1 shows regular and MeSH keywords for literature search.

**Table 1:** Regular and MeSH keywords for literature search.

Regular keyword-STEMI	
Total Records	22691
Records selected	1800

Regular keyword- STEMI Major Adverse Cardiovascular Events	
Total records	1277
Records selected	178

MeSH Keyword STEMI (Subheading- Complications)	
Total records	276
Records selected	87

Studies were selected after applying the following Inclusion/Exclusion Criteria

Inclusion Criteria

1. Human subjects of age 45+ years

2. Diagnosis of STEMI have positive EKG

3. Paper published in English language and within the past 5 years

4. The study types were observational studies, clinical trial including randomized controlled trial, cohort study, case-control study or review article

5. All full papers

Exclusion Criteria

1. Animal Studies

2. Non- English language literature

3. Meta-analysis, case report and case series study

Results

Table 2 shows the total number of articles after applying inclusion/exclusion criteria in the following order

**Table 2:** Total number of articles after applying inclusion/exclusion criteria

Regular keyword-STEMI	
Total Records	22691
Inclusion/Exclusion	
Humans	19128
English Language	17286
Published Within 5 years	6066
Patient Age 45+ years	4518
Full Text online	1800

Regular keyword- STEMI Major Adverse Cardiovascular Events	
Total records	1277
Inclusion/Exclusion	
Humans	1074
English language literature	1031
Published within 5 years	529
Patient age 45+ years	455
Full Text online	178

MeSH Search MeSH Keyword STEMI (Subheading - Complications)	
Total records	276
Inclusion/Exclusion	
Humans	274
English language literature	259
Published within 5 years	259
Age 45+ years	216
Full Text online	87

A total of 1622 articles from keyword search ‘STEMI’ were excluded due to lack of outcome of interest “Major Adverse Cardiovascular Events” and removal of duplicates. After a refined search, the total number of articles obtained was 178 free full texts. All 178 free full texts were reviewed and 103 were removed due to one of the following reasons:

- Not specifying the disease of interest (those which did not assess for STEMI separately but were rather a composite assessment of STEMI with NSTEMI or ACS as a whole or both)

- Case Report or Case Series Studies (as it only assessed for a particular patient in focus)

- Meta-analysis

- Data Extraction not possible by quality assessment.

 

Finally, 75 publications in PubMed (with free full text available online) were reviewed, which included:

- 51 observational studies, among which one specifically identified itself as a prospective observational study.

- Five studies that identified themselves as RCT and eight other studies that identified themselves as clinical trials [7-19].

- Eight studies identified as Cohort (including two identified as a retrospective cohort and five identified as a prospective cohort) [20-27].

- One study identified as a cross-sectional study (n=1244)

-one as case-control study and

-one as a review article [28-30].

The maximum number of subjects in a study was 15,628 and the minimum was 8, and the total number of subjects included in all 75 studies was 77,782 [31,17]. Among all 75 studies, 62 studies explained percutaneous coronary intervention (PCI) either as the intervention of choice or as a primary intervention inclusive of other management strategies. Coronary angiography was explained as the investigation of choice in four studies [9,32-34].

All the records reviewed were freely available for review and the citations for the borrowed definitions are available. A qualitative review was performed on the available records after Inclusion/Exclusion to include the relevant disease and population with the required outcome.

**Figure 1:**
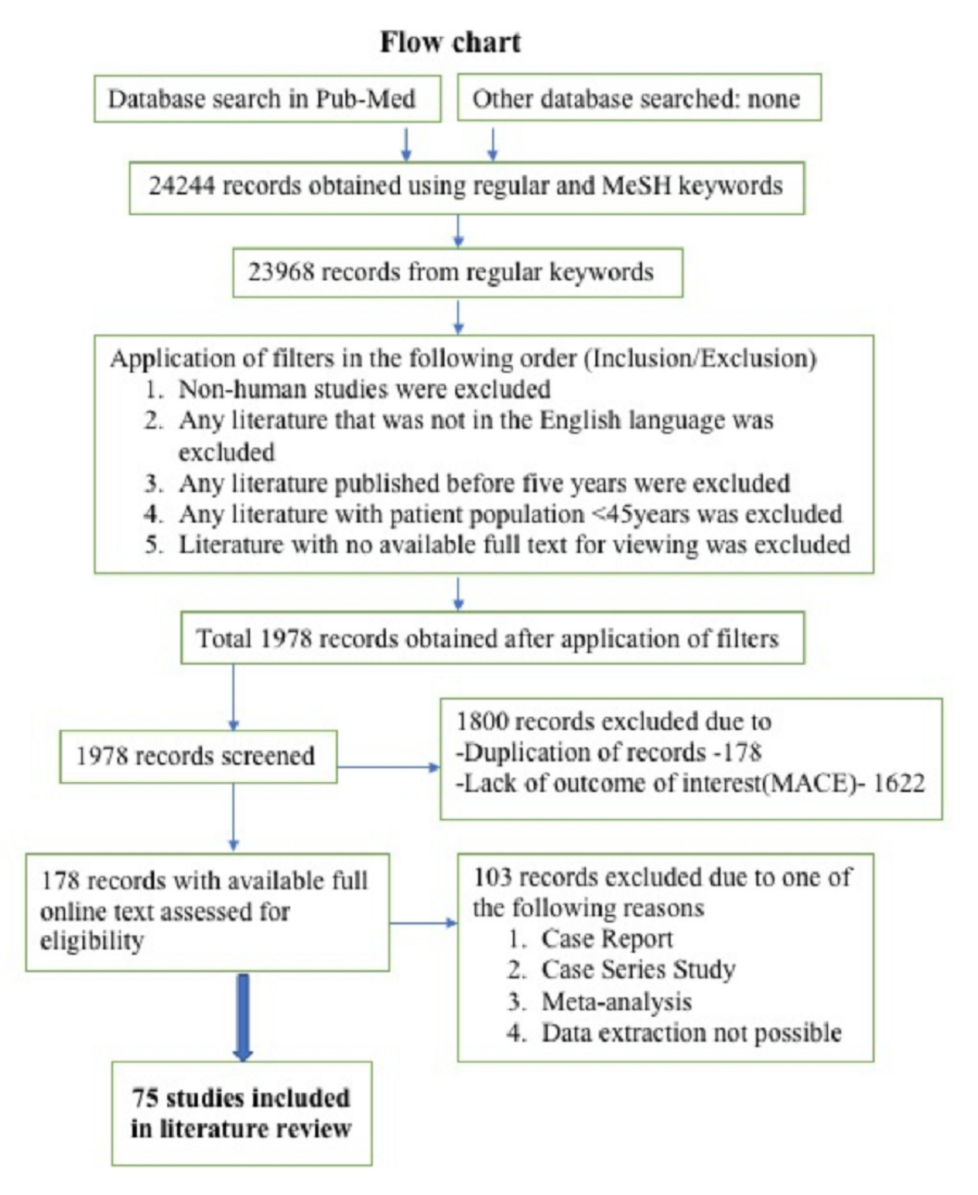
Flow chart showing the process of current literature review

The figure below shows the flowchart with the process of current literature review (figure 1).

Discussion

The analysis performed was aimed at demonstrating how STEMI was related to MACE irrespective of the management strategy. Though MACE was observed with all the modalities of management for STEMI, the strength of association was not assessed. We firmly believe that there are large variations in the number of MACE events when clearly analyzed for different modalities of treatment. We also found out that MACE incidence depends upon the pre-STEMI health status, age, gender, race, co-morbidities of the patient and many other factors which are not yet explored.

The endpoint of study was major adverse cardiovascular outcome which was explained in the reviewed literature as combination of at least one or more of: all-cause mortality/death (37 studies), re-infarction (25 studies), cardiovascular mortality/death (23 studies), repeat revascularization (18 studies), stroke (16 Studies), heart failure (14 studies), non-fatal re-infarction (11 studies), stent thrombosis (eight studies), major bleeding (seven studies), microvascular obstruction (five studies), re-hospitalization for cardiovascular-related illness (four studies), repeat PCI (four studies), non-cardiovascular mortality/death (two studies) and transient ischemic attack (one study)[7-45].

Table 3 summarized some of the studies with MACE reported from selected data for the literature review:

**Table 3:** Summary of some of the studies with MACE reported from selected data for the literature review.

Author/ Date	Study Design	Population with STEMI	Sample Size	Main Points	p-value
Lee et al. [35],2017	Observational Study	363 patients with anemia and rest of them with no anemia (between 2005-2014)	1751	MACE was 33.8% vs 22.9 % in anemia and non-anemia group respectively	P<0.001
Liu et al. [36],2015	Observational Study	Follow up with serum apelin levels for patients who underwent PCI	120	34.3% patients in the low apelin group compared to 13.3% in high apelin group had MACE	N/A
Li et al. [28],2017	Multicenter Cross-Sectional Study	607 patients (June 2009 - June 2010) and 637 patients (2015) from hospitals in Northeast China	1244	No significant change in MACE [13.34% vs. 13.66%] in 5 years	P = 0.872
Yu et al. [37],2017	Observational Study	Patients who underwent PCI with a mean age of 59.1 years	323	MACCE occurred in 38 patients (12%)	N/A
Cheng et al. [27],2014	Cohort Study	Patients treated with primary PCI followed by measurement of triglyceride (TG)	247	The fewer occurrence of MACE with lower TG compared to higher TG levels (26.1% vs. 11.9%)	p = 0.0137
Grundeken et al. [38],2017	Observational Study	Patients with bifurcation (n=123) and non-bifurcation (n=842) lesion undergoing PCI with self-apposing-stents.	965	MACE (8.7% vs. 8.4%) in bifurcation vs. non-bifurcation lesion.	N/A
Reinstadler et al. [13],2016	Clinical Trial	792 STEMI patients re-perfused within 12 hrs. of symptom onset followed up for 12 months for MACE which included 540 (68%) patients with antecedent hypertension	792	MACE with hypertensive patient vs non-hypertensive was [45 patients vs eight patients]	p-value <0.01
Nakashima et al. [39],2017	Observational Study	Patients with primary PCI including 212 patients with MI onset in the morning.	663	MACE was higher with morning onset of MI compared to other MI onset at other time [21% vs 4%]	p=0.012
Li et al. [40],2018	Observational Study	Patients with primary PCI with Drug-Eluting Stent either with Trans-Radial Intervention(TRI) or with Trans Femoral Intervention(TFI)	689	After propensity score matching the incidence of MACE was TFI > TRI [11.6% vs. 4.6%]	p-value of 0.018
Park et al. [41],2016	Observational Study	Patients with STEMI from INTERSTELLAR STEMI registry who underwent PCI were analyzed for follow up period of 2.2±1.6 years	668	MACCE 14.1% (9.7% MACCE and 5.2% all-cause mortality)	N/A
Kołtowski et al. [7],2016	Randomized Control Trial	Patients from OCEAN trial undergoing PCI with radial (n=52) vs. femoral (n=51) approach.	103	In radial vs. femoral group [9.6% vs. 11.8%]	p=0.48
Reinstadler et al. [42],2016	Observational Study	Patients undergoing primary PCI followed up for specific period.	200	10% suffered MACE.	p=0.001
Rajesh et al. [44],2018	Observational Study	Follow up for 314 among total patients who underwent PCI with very long Drug Eluting Stent	343	MACE was observed in 6% patients	N/A
Lønborg et al. [45],2014	Observational Study	Patient who underwent PCI. ST peak was analyzed for every patient.	942	ST peak was associated with a higher rate of MACE [26.9% vs. 18.2%]	p=0.002

Due to the widespread use of PCI, and least number of papers published with other modes of management as the primary treatment modality for STEMI, the study could not explore much in areas of specific management strategies. MACE occurrence following STEMI is unpredictable, but the rate of occurrence could be minimized with appropriate treatment approach and strategy. More studies are needed to analyze the outcomes of different management strategies in lowering the incidence of MACE. Even with new advanced techniques and technologies, comparisons between the new and old strategies in management should be done in order to find out both long and short-term outcomes.

## Conclusions

The objective of our study is to review the relationship between STEMI and major adverse cardiovascular events irrespective of the treatment modality. The current literature review concluded that MACE remains one of the strongest adverse outcomes in STEMI patients. The incidence of MACE ranges from 4.2% to 51% irrespective of the mode of treatment, with follow-up visits ranging from day 0 to 10 years following STEMI. The current literature review has some limitations: the study limits its analysis in terms of age (patients involved were ≥45 years old), gender (no gender-specific analysis was performed), modality of treatment, duration of follow up (none of the literature explained the long term follow up ≥10 years) and many other unexplored factors which can be tested in future studies.
